# Building thalamic neuronal networks during mouse development

**DOI:** 10.3389/fncir.2023.1098913

**Published:** 2023-02-03

**Authors:** Irene Huerga-Gómez, Francisco J. Martini, Guillermina López-Bendito

**Affiliations:** Instituto de Neurociencias de Alicante, Universidad Miguel Hernández-Consejo Superior de Investigaciones Científicas (UMH-CSIC), Sant Joan d’Alacant, Spain

**Keywords:** thalamus, development, mouse, thalamocortical, interneurons

## Abstract

The thalamic nuclear complex contains excitatory projection neurons and inhibitory local neurons, the two cell types driving the main circuits in sensory nuclei. While excitatory neurons are born from progenitors that reside in the proliferative zone of the developing thalamus, inhibitory local neurons are born outside the thalamus and they migrate there during development. In addition to these cell types, which occupy most of the thalamus, there are two small thalamic regions where inhibitory neurons target extra-thalamic regions rather than neighboring neurons, the intergeniculate leaflet and the parahabenular nucleus. Like excitatory thalamic neurons, these inhibitory neurons are derived from progenitors residing in the developing thalamus. The assembly of these circuits follows fine-tuned genetic programs and it is coordinated by extrinsic factors that help the cells find their location, associate with thalamic partners, and establish connections with their corresponding extra-thalamic inputs and outputs. In this review, we bring together what is currently known about the development of the excitatory and inhibitory components of the thalamocortical sensory system, in particular focusing on the visual pathway and thalamic interneurons in mice.

## Introduction

The thalamus has classically been considered a relay station in the brain due to its central location and patterns of connectivity. Excitatory neurons in sensory nuclei receive ascending information from peripheral organs and they project their axons beyond the thalamus, mainly into the sensory cortices and avoiding intrinsic connections (Petersen, 2007; Huberman et al., 2008; Tsukano et al., 2017). As the vast majority of cells in sensory nuclei are excitatory neurons, the thalamus could be considered to be merely a relay station of sensory information, transferring messages from the periphery to the cortex. However, there is an increasing body of evidence demonstrating a key role of thalamic nuclei in processing information and gating messages to the cortex. In addition to the ascending sensory information, excitatory neurons integrate signals from other brain structures and from the intrinsic thalamic networks. GABAergic neurons represent an important element in these intrinsic networks and despite their small number, the GABAergic neurons in the interconnected networks shape the output of the sensory thalamus (Hirsch et al., 2015).

Thalamic circuits develop progressively in embryonic stages and they finally assemble during postnatal life (Jhaveri et al., 1991; Schlaggar and O’Leary, 1994). Excitatory neurons are born in the ventricular zone of the developing thalamus, thereafter migrating toward the mantle zone where they start to extend dendrites and axons. Along their route toward the cortex, the axons of excitatory neurons project through different brain territories, bundling into fascicules, branching and making synaptic connections (Crowley and Katz, 2000; Hannan et al., 2001; Hevner et al., 2002; López-Bendito and Molnár, 2003; Gurung and Fritzsch, 2004; Hensch, 2004; Pfeiffenberger et al., 2005; Miko et al., 2008; Hanganu-Opatz, 2010). By contrast, the spatiotemporal developmental trajectory of local GABAergic neurons differs considerably. These GABAergic neurons are not derived from thalamic progenitors but rather, they are born and migrate from neighboring midbrain and pre-thalamic domains, invading the thalamus and integrating into its circuits some time after excitatory neurons. In this review, we bring together what is currently known about the development of the excitatory and inhibitory components of the thalamocortical sensory system, focusing particularly on the visual pathway and on thalamic interneurons in mice.

## The functional organization of the sensory thalamus

**FIGURE 1:**
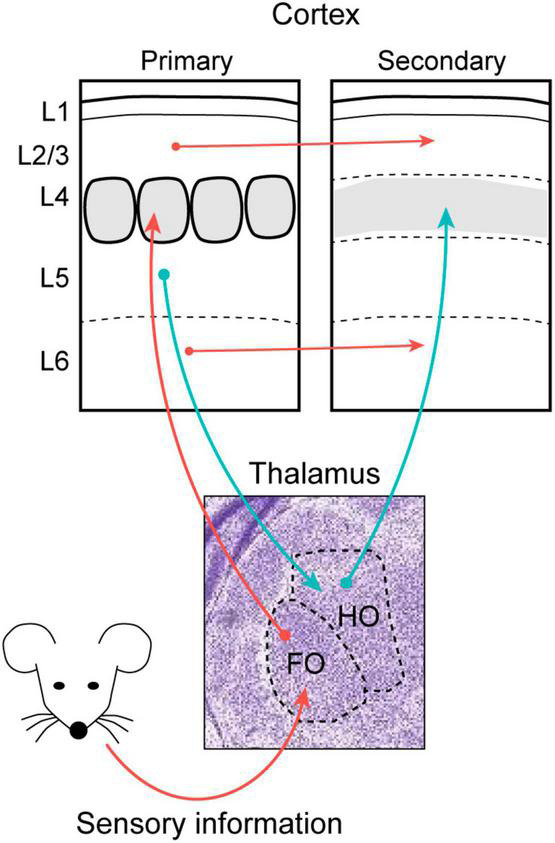
The thalamus transmits peripheral sensory information to primary sensory areas and retransmits the processed sensory message to secondary cortical areas. The schema represents the major feed-forward connections of the mouse whisker system. Stimuli from peripheral sensory organs arrive at first order thalamic nuclei (FO) which connects with L4 neurons in the primary sensory cortex. The information then spreads to other cortical areas both through direct cortico-cortical connections (red arrows) and through the trans-thalamic pathway using higher-order thalamic nuclei (HO) as relay stations (blue arrows).

Thalamic neurons are organized into spatial clusters or nuclei that can be characterized through the subcortical origin of their afferents. Some of these afferents carry sensory information derived from peripheral organs, which adopt a modal organization to define the primary sensory nuclei of the thalamus: the dorsolateral geniculate nucleus (dLG) that receives visual information; the ventral posteromedial nucleus (VPM) for somatosensory input; and the ventral medial geniculate nucleus (MGv) for auditory input. While the dLG, VPM and MGv are classified as first-order (FO) nuclei since their main driving stimuli arrive directly or indirectly from the peripheral sensory organs (Sherman, 2017; Halassa and Sherman, 2019), the thalamus also contains higher-order (HO) nuclei that receive driving inputs from subpopulations of projection neurons in Layer 5b (L5b) of the respective cortical areas (Sherman and Guillery, 2002; Bickford, 2016). HO nuclei mainly process unimodal information, even though they can integrate inputs from other modalities too. Among the HO nuclei, the lateral posterior nucleus (LP, visual), posterior medial (PoM, somatosensory), and the dorsal medial geniculate nucleus (MGd, auditory) not only further process sensory information but they also help the thalamus to connect different cortical areas (Figure 1; Butler, 2008; Halassa and Sherman, 2019).

The patterns of connectivity between the thalamus and cortex are similar for the different sensory modalities. Thus, sensory stimuli ascending from the peripheral organ arrives at the corresponding FO nucleus, which in turn projects to the L4, L5b, and L6 of the corresponding sensory cortex (Swadlow and Alonso, 2017). Within a cortical column, information flows from L4 to L2/3 and from there to L5 and L6, the layers that project out of the cortex. Neurons from L5b and L6b send projections to HO nuclei, and neurons in L6a project back to the FO nuclei (Sumser et al., 2017; Hoerder-Suabedissen et al., 2018). Therefore, these connections form feedback and feedforward loops that establish the basis of sensory processing in the thalamocortical system (Viaene et al., 2011). As a result, the thalamus represents a hub that can send information to and from different cortical areas (Sherman and Guillery, 2002; Reichova and Sherman, 2004; Lee and Murray Sherman, 2010; Theyel et al., 2010).

## The formation of the thalamocortical system

### Patterning of the diencephalon

**FIGURE 2:**
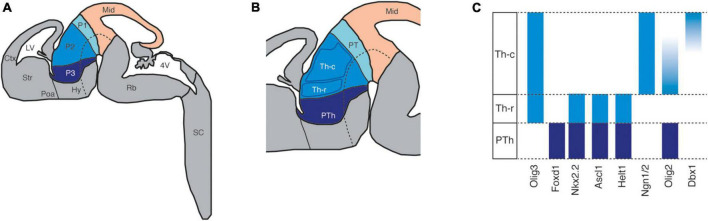
The patterning of the developing diencephalon. **(A)** The schema represents a sagittal view of the developing mouse brain highlighting the midbrain (Mid) and the subdivisions, known as prosomeres (P1, P2, and P3), of the diencephalon according to the prosomeric model. Ctx: cortex; Hy: hypothalamus; LV: lateral ventriculus; Poa: preoptic area; Rb: rhombencephalon; SC: spinal cord; Str: striatum; 4V: fourth ventriculus. **(B)** The two progenitor domains of the prosomere 2 or thalamus (Th): Th-c (caudal) and Th-r (rostral). PTh: prethalamus; PT: pretectum. **(C)** Representative expression patterns of transcription factors in the different the progenitor domains of prosomeres 2 and 3.

Early in development, the diencephalon subdivides into three transverse regions called prosomeres, each of which can be further subdivided into four longitudinal bands or plates: roof, alar, basal, and floor (in a dorsoventral order, Figure 2A). This percolation relies on the presence of gradients of diffusible molecules, such as the wingless-INT proteins (WNTs), bone morphogenetic proteins (BMPs), Sonic hedgehog (SHH), and fibroblast growth factor proteins (FGFs) (Kataoka and Shimogori, 2008; Martinez-Ferre and Martinez, 2009, 2012). Prosomeres are evident at E10 in mice and their respective alar plates develop into different brain structures: prosomere 1 gives rise to the pretectum, which includes multiple domains of the adult brain that are involved in processing visual information and in the execution of visual reflexes (Ferran et al., 2009); prosomere 2 develops into the epithalamus and thalamus; and lastly, prosomere 3 gives rise to the prethalamus, which includes GABAergic structures like the reticular nucleus (RTN) and the zona incerta (ZI) (Puelles and Rubenstein, 2003).

Prosomeres 2 and 3 are separated by the zona limitans intrathalamica (ZLI), an organization center that expresses high levels of SHH. During the early percolation of the diencephalon, SHH is a fundamental signaling molecule in prosomere 2 because it steers the differentiation of the prospective thalamus from the epithalamus (Chatterjee and Li, 2012; Chatterjee et al., 2014; Mallika et al., 2015). In the prospective thalamus, two progenitor zones are established by E10.5 in the mouse (Figure 2B): the rostral and the caudal progenitor domains (Jeong et al., 2011; Suzuki-Hirano et al., 2011). The progenitors in the rostral domain are exposed to higher concentrations of SHH and consequently, they express markers like *Olig3, Nkx2.2, Ascl1*, and *Olig2* (Scholpp et al., 2009). On the other hand, the progenitors in the caudal domain are exposed to less SHH, such that they express markers like *Olig3, Ngn1/2*, and *Dbx1* (Figure 2C; Barth and Wilson, 1995; Hashimoto-Torii et al., 2003; Kiecker and Lumsden, 2004; Scholpp et al., 2006; Szabó et al., 2009; Vue et al., 2009). Neurogenesis in these progenitor domains spans approximately from E10 to E13 (Wong et al., 2018) and the postmitotic progeny differentiate into two broad different cell types: the caudal domain differentiates into the glutamatergic projection neurons that populate the thalamic nuclei and form thalamocortical connections (Tou et al., 2007; Price et al., 2012), and the rostral domain differentiates into GABAergic projection neurons that populate the intergeniculate leaflet (IGL) and the perihabenular nucleus (pHB) (Delogu et al., 2012; Fernandez et al., 2018).

As most neurons in the adult thalamus derive from the caudal progenitor domain, they must undergo a complex process of specification to generate neuronal diversity and the distinct thalamic nuclei. Although it is not clear how this diversification occurs, some evidence indicates that the population of caudal progenitors is heterogeneous. Within this caudal progenitor domain, there are transcription factors that are expressed in opposite gradients through the antero-ventral to caudo-dorsal axis. For instance, the expression of *Olig2* is high at the most anterior pole and the expression of *Dbx1* in the most caudal pole. This polarization of the caudal progenitor domain has relevant implications for thalamic nucleogenesis. Lineage and birth-dating analysis demonstrated that antero-ventral progenitors give rise to neurons populating more latero-ventral nuclei, and caudo-dorsal progenitors to more medio-dorsal ones (Tou et al., 2007; Wong et al., 2018). In addition, to this spatial order, there is also a temporal sequence whereby the formation of the latero-ventral nuclei occurs earlier than the medio-dorsal nuclei, which is consistent with the earlier transition from symmetric to asymmetric division in the antero-ventral compared to the caudo-dorsal progenitors (Nakagawa and Shimogori, 2012; Wong et al., 2018).

If different subpopulations of progenitors give rise to diverse cell types populating thalamic nuclei, it is expected that they are controlled by different genetic programs. However, data from single-cell RNA sequencing of E12 mice suggests that the progenitors in the ventricular zone of the caudal domain (apical progenitors) comprises a unique cluster of cells based on their transcriptomic profile. There is, indeed, a second cluster of dividing cells that derives from the apical progenitors and corresponds to the basal (or intermediate) progenitors of the thalamus located away from the ventricular zone (Guo and Li, 2019). Apical progenitors generate larger clones than basal progenitors, 12 neurons on average, and both apical or basal-derived clones tend to occupy more than one nucleus (Wong et al., 2018). It has been suggested that sibling cells tend to occupy functionally related nuclei but further evidence is needed (Shi et al., 2017). Despite transcriptomic analysis does not reveal clear-cut internal subdivisions of progenitor domains, they do show a graded pattern of gene expression (Guo and Li, 2019), as previously observed in data obtained using labeling methods (Tou et al., 2007). In sum, to refine the classification of the apparently heterogeneous populations of progenitors in the caudal domain of the thalamus, it is necessary to generate larger databases of single-cell transcriptomic profiles, accompanied by more complete atlases of gene expression and sophisticated algorithms.

**FIGURE 3:**
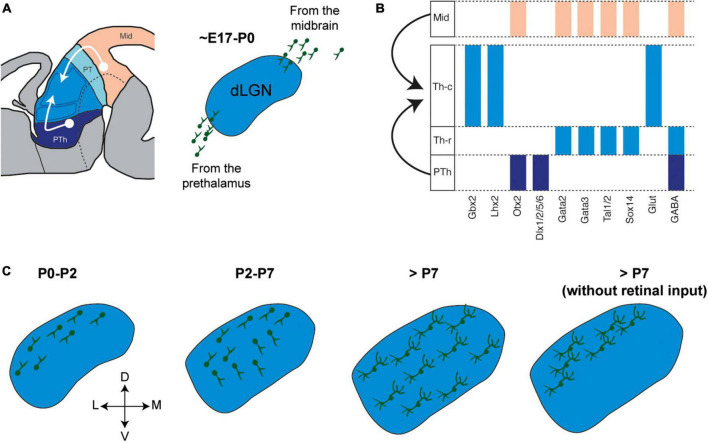
Integration of local thalamic interneurons from extra-thalamic sources. **(A)** *Right*, Schema showing the migration toward the thalamus of prospective local interneurons born in the midbrain and prethalamus. *Left*, Prospective local interneurons start invading the developing mouse dLGN shortly before birth. **(B)** Representative expression patterns of transcription factors, specially lineage selector genes, in precursor cells from prosomere 2 and GABA lineages from prosomere 3 and midbrain. **(C)** Schema of a mouse dLGN showing sequentially how local interneurons migrate and distribute into the nucleus in control conditions and upon deprivation of retinal input (Golding et al., 2014; Charalambakis et al., 2019; Su et al., 2020).

Although the projection neurons of thalamic nuclei are born in the caudal proliferative domain, it remains unclear whether their nucleus-specific identity is specified at the progenitor stage (Chatterjee et al., 2014). Rather, current evidence suggests that nucleus-specific identity is conferred when progenitors exit the cell-cycle. The gradual expression of the *Gbx2* transcription factor by post-mitotic cells is one determinant of thalamic nuclei that project to the cortex (Figure 3B). The expression of *Gbx2* starts at E9.5, following a very dynamic spatiotemporal pattern that ultimately defines the borders of the thalamus with the epithalamus, prethalamus and pretectum, as well as parcellating the thalamus into distinct nuclei (Nakagawa and O’Leary, 2001; Tou et al., 2007; Chen et al., 2009; Li et al., 2012). The absence of *Gbx2* leads to a shrinking of the thalamus at its posterior and dorsal borders, enlarging the pretectum and epithalamus, respectively. In turn, there is a disruption in the histogenesis of the nuclear complexes and a significant loss of thalamocortical projections (Chen et al., 2009; Chatterjee and Li, 2012; Mallika et al., 2015; Nakagawa, 2019).

### The development of thalamocortical projections

**FIGURE 4:**
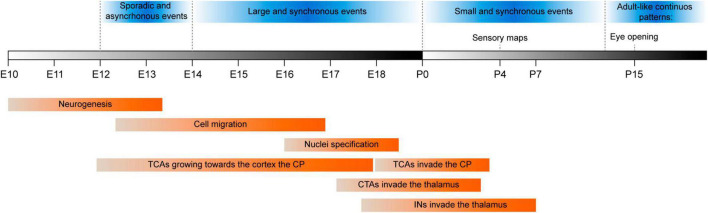
Timeline showing milestones in mouse thalamic development (red boxes). The different patterns of spontaneous thalamic activity throughout development (blue boxes). First, thalamic spontaneous activity is characterized for being scarce and the events are uncorrelated (E12-E14). This is followed by waves of spontaneous activity (E14-P0) that propagate among FO and then HO nuclei. At perinatal stages, these waves disappear and instead, there are small events most likely triggered spontaneously from peripheral organs that engage cortical areas. Before eye opening, there is a switch to an adult-like pattern where spontaneous activity becomes dense, asynchronous and continuous (Colonnese et al., 2010). CP, cortical plate; CTAs, corticothalamic axons; E, embryonic day; Ins, interneurons; P, postnatal day; TCAs, thalamocortical axons.

In the developing mouse thalamus, prospective thalamocortical cells begin to extend their axons toward the cortex at around E12, shortly after neurogenesis ceases (Figure 4). Growing thalamocortical axons are guided through different territories by molecular and cellular cues, as well as by activity-dependent mechanisms (Marcos-Mondéjar et al., 2012; Mire et al., 2012; Molnár et al., 2012; Leyva-Díaz et al., 2014; Castillo-Paterna et al., 2015). Thalamocortical axons start their journey toward the cortex by growing rostrally through the thalamus and heading toward the prethalamus. They navigate through the thalamic territory using prethalamic axons as scaffolds and following gradients of guidance cues, such as Sema3a and Netrin1 (Quintana-Urzainqui et al., 2020). Thalamocortical axons traverse the entire prethalamus and move through the Slit-free domain of the peduncular hypothalamus until they encounter the diencephalic-telencephalic boundary (DTB) (Callejas-Marin et al., 2022). Slit proteins guide thalamocortical axons out of the hypothalamus and they prevent them from crossing the midline (Bagri et al., 2002; López-Bendito et al., 2007; Braisted et al., 2009; Bielle et al., 2011). Subsequently, thalamocortical axons cross the DTB, attracted by Netrin1, and they enter the ventral telencephalon (Métin and Godement, 1996; Braisted et al., 2000).

Thalamic projections continue their journey through the telencephalon, reaching the internal capsule from where they follow a permissive corridor formed between two repulsive areas: the proliferative zone of the medial ganglionic eminence (MGE) and the globus pallidus. A large proportion of these corridor cells are GABAergic neurons derived from the lateral ganglionic eminence (LGE), cells that express membrane-bound Neuregulin1 and that migrate into the mantle of the MGE between E11-E14 (López-Bendito et al., 2006; Bielle et al., 2011). The topographic organization of thalamocortical axons is preserved throughout the ventral telencephalon, which is a result of complex interactions between cues like Netrin1, Sema3a/3f, Slit1, L1 cell-adhesion molecules and ephrinA5 (Dufour et al., 2003; Seibt et al., 2003; Mire et al., 2012; Leyva-Díaz et al., 2014; Castillo-Paterna et al., 2015).

By E14, thalamocortical axons meet corticofugal axons at the pallial-subpallial boundary (PSPB), and they use them as scaffolds to turn into the pallium and spread across the developing cortex (McConnell et al., 1989; Blakemore and Molnar, 1990; de Carlos and O’Leary, 1992). The timing of the arrival of thalamic axons is relevant for cortical development. As thalamic inputs arrive at a rather immature cortex, they can exert a major effect on ongoing processes such as neurogenesis, migration and differentiation (Cadwell et al., 2019). Thalamocortical axons start to invade the cortical plate at E17 when the granular layers are being formed (Allendoerfer and Shatz, 1994; del Río et al., 2000; Molyneaux et al., 2007; Little et al., 2009), and they finally reach their destination during the first postnatal week (Figure 4), mainly targeting neurons in L4 and to a lesser extent, neurons in L5b (López-Bendito and Molnár, 2003).

While invading the cortical plate, thalamic axons form functional synapses with subplate cells, a transient layer of rather mature neurons located below the cortical plate that coordinate the early maturation of thalamocortical networks (Kanold and Luhmann, 2010; Hoerder-Suabedissen and Molnár, 2015). As Cajal-Retzius cells, another transient population of cortical neurons, subplate neurons disappear by programmed cells death during the first postnatal days in mouse and thalamocortical afferents form direct contacts with neurons in L4 and deeper layers. Apart from subplate neurons, thalamocortical afferents form transient circuits with a specific subpopulation of developing interneruons. During the first postnatal week in mice, thalamocortical axons contact L5 somatostatin-positive interneurons that in turn contact spiny stellate neurons in layer 4 (Marques-Smith et al., 2016). This circuit becomes remodeled and disappears by the end of the first postnatal week. Despite its brief duration, the connection between L5 somatostatin-positive interneurons and L4 neurons orchestrates the assembly of local inhibition in layers 4. Also the density of thalamocortical input to infragranular interneurons varies during development. Somatostatin-positive interneurons receive a transient strong thalamic drive at immature stages that is required for the correct assembly of thalamic feed-forward inhibition mediated by parvalbumin-positive interneurons (Tuncdemir et al., 2016).

### The development of corticothalamic projections

In mice, corticothalamic projections appear at E10 from post-mitotic neurons in the cortical plate. These corticofugal projections navigate laterally through the intermediate zone until they reach the PSPB between E13 and E15 (Jacobs et al., 2007). At this boundary, corticothalamic axons interact with the ascending thalamocortical axons, facilitating their invasion of the cortical territory. The corticothalamic axons then continue their journey through the ventral telencephalon toward the internal capsule, where molecular cues and corridor cells guide them toward the diencephalon (Bagri et al., 2002; López-Bendito et al., 2006, 2007). By E15, corticothalamic axons have crossed the DTB to enter into the prethalamus, where more-or-less a day later they interact with cells from the RTN and the perireticular nucleus (PRN) (Garel and Rubenstein, 2004; Molnár et al., 2012; Deck et al., 2013). These axons are sorted in the prethalamus and the majority of axons from L5 are directed toward the cerebral peduncle, while axons from L6 and the remaining axons from L5 are directed toward their thalamic targets (Clascá et al., 1995; Molnár and Cordery, 1999; Jacobs et al., 2007). Mouse corticothalamic axons invade the thalamus just prior birth (Figure 4), first entering the developing somatosensory nuclei, and then invading the auditory and visual nuclei, which are fully innervated by the end of the first postnatal week (Jacobs et al., 2007; Grant et al., 2012). However, the cellular and molecular mechanisms that guide the entry of corticothalamic axons into the thalamus remain poorly understood. Nevertheless, removal of retinal input alters corticothalamic innervation of the dLGN, inducing premature entry of L6 axons and an abnormal cross-hierarchical invasion of L5 axons that would otherwise be designated to the LP (Brooks et al., 2013; Grant et al., 2016; Moreno-Juan et al., 2022).

### Early thalamic inputs affect cortical development and specification

The thalamus and cortex develop at a relatively similar pace and consequently they may influence each other’s maturation. The thalamus clearly affects many aspects of cortical development, such as the radial organization, cell proliferation, specification of cortical areas, navigation of corticothalamic axons, interneuron maturation and circuit assembly (Rakic, 1991; Dehay et al., 1996; Zechel et al., 2016). More specifically, glutamate released by thalamocortical axons is required for Reelin-expressing cortical interneurons to develop (de Marco García et al., 2015). Similarly, thalamic inputs regulate the integration of somatostatin- and parvalbumin-expressing interneurons into cortical circuits (Wamsley and Fishell, 2017), and the segregation of pyramidal neurons in the L4 of the barrel cortex (Li et al., 2013; Assali et al., 2017). Thalamic input also affects cell identity in the cortex, since differentiation into primary or higher-order cortical areas relies on the arrival of thalamocortical axons (Chou et al., 2013). Indeed, when FO nuclei are removed genetically, primary sensory areas acquire the molecular and functional properties of secondary cortical areas (Chou et al., 2013; Vue et al., 2013; Pouchelon et al., 2014). Recently, somatostatin-expressing interneurons in the cortex were seen to be necessary for the correct arrival of thalamocortical inputs onto parvalbumin-expressing interneurons and pyramidal neurons during the first week of postnatal life in mice (de Marco García et al., 2015; Marques-Smith et al., 2016; Tuncdemir et al., 2016; Che et al., 2018; Takesian et al., 2018).

Neuronal transmission in the thalamocortical system can also influence the development of the cortex, as seen when normal synaptic transmission is disrupted in knock-out mice that lack key synaptic proteins, such as NMDA receptor 1, adenylyl cyclase 1, or metabotropic glutamate receptor 5. Such disruption of neurotransmission can provoke a lack of neuronal organization and smaller barrels with blurry borders in S1 (Iwasato et al., 2000; Datwani et al., 2002; Ballester-Rosado et al., 2010; Antón-Bolaños et al., 2019).

## Spontaneous activity in the developing thalamus

Neuronal activity in the developing thalamus evolves over different stages in mice (Figure 4). In a first stage, endogenous and uncorrelated activity spans mid-embryonic mouse development (E12-E14), which affects the expression of genes involved in thalamocortical axon growth and branching when manipulated (Herrmann and Shatz, 1995; Uesaka et al., 2007; Mire et al., 2012; Castillo-Paterna et al., 2015; Moreno-Juan et al., 2017). After this initial stage, the activity in the thalamus becomes more synchronous and by E14, spontaneous synchronic activity takes the form of waves of spontaneous activity that initially propagate through FO nuclei and that later also engage HO nuclei (Moreno-Juan et al., 2017). After birth, spontaneous activity becomes less correlated, especially in the somatosensory and auditory nuclei and at P2, in the visual nucleus as well (Colonnese et al., 2010). The waves of spontaneous activity observed in the thalamus are transmitted along thalamocortical axons to the developing cortical areas and consequently, early thalamocortical input could have an impact on cortical development through activity-dependent mechanisms. Indeed, altering patterns of activity through genetic manipulation provokes cross-modal changes in the development of sensory areas in the cortex (Moreno-Juan et al., 2017; Antón-Bolaños et al., 2018). The electrical properties of the thalamocortical circuit progressively mature during the first two postnatal weeks, undertaking more continuous and decorrelated spontaneous firing (Murata and Colonnese, 2016, 2018; Martini et al., 2021). This transition in spontaneous thalamic activity seems to be critical for the onset of the active processing of environmental information by the cortex. Moreover, it might be caused by changes in the sensory organs, synaptic maturation or circuit remodeling, such as the gradual integration of inhibitory components (Demas et al., 2003; Colonnese, 2014; Sokhadze et al., 2018).

## Thalamic interneurons

### General overview

Thalamic neurons receive inhibitory inputs from projecting neurons residing in the prethalamus (RN, ZI, and vLGN) and from other extra-thalamic sources, such as the superior colliculus, basal ganglia, hypothalamus and pontine reticular formation (Halassa and Acsády, 2016). In addition, they are also inhibited by local GABA-releasing neurons, although the number and distribution of these local interneurons is not conserved across species. In small mammals like mice, marsupials and bats, interneurons are sparse and mainly found in the dLGN, whereas they are abundant and widely distributed throughout the thalamus in large mammals. Inhibitory interneurons are absent from the dLGN of some non-mammalian amniotes, such as crocodiles, lizards and snakes, but they are present in birds (Butler, 2008). Local interneurons are not the only GABAergic cells in the mature thalamus, since there is a small subpopulation of GABAergic cells that reside within the IGL and pHB whose axons project to extra-thalamic targets (Harrington, 1997; Tou et al., 2007; Delogu et al., 2012; Inamura et al., 2012; Fernandez et al., 2018).

### The origin of thalamic interneurons

Local interneurons that integrate into thalamic circuits are not born in the proliferative zone of the developing thalamus but rather, they migrate into the thalamus from other brain regions. Across species, some regions that generate thalamic neurons are conserved but also, additional regions are observed as thalamic circuits increase in complexity and size. The midbrain proliferative zone generates a stream of cells that colonizes the developing thalamus and that is made up of cells that differentiate into local GABAergic cells (Figure 3A; Jones, 2002; Hayes et al., 2003; Bakken et al., 2015; Jager et al., 2021). In the mouse, the invasion of these local interneuron precursors begins at E17, starting from the caudal tier of the developing thalamus. Fate mapping experiments confirmed the midbrain origin of these cells, showing that they are born at approximately E10-E13 and that they belong to the *Engrailed1* lineage (Jager et al., 2016, 2021), a transcription factor that is expressed in the midbrain and not the forebrain (Sgaier et al., 2007). The precursors generated from the *Engrailed1* progenitors are also characterized by the expression of the transcription factors *Sox14, Gata2*, and *Otx2* (Figure 3B). Once in the thalamus, these midbrain-derived interneurons adopt a specific spatial distribution, whereby they are enriched in FO nuclei but they also appear in HO and rostral nuclei (Jager et al., 2021). This subpopulation of GABAergic cells constitutes the largest of the local interneuron populations in the mature thalamus.

Another source of thalamic interneurons in the mouse is the developing prethalamus or prosomere 3 (Figure 3A). Located rostral to the thalamus, the developing prethalamus generates several GABAergic cell lineages, most of which populate prethalamic structures like the RTN and the vLGN, while others establish a stream of cells that invade the developing thalamus from its rostral tier around the time of birth (Golding et al., 2014; Jager et al., 2021). Approximately 20% of the population of local interneurons in the mature thalamus are specified in the developing prethalamus from a lineage that expresses *Dlx1/2, Foxd1*, and *Dlx5/6*, and that does not express *Lhx6* or *Nkx2.1* (Figure 3B). The prethalamus-derived interneurons that will invade the thalamus have features complementary to midbrain-derived interneurons, for example, they do not express *Sox14* and they are enriched in HO nuclei (Jager et al., 2021).

Studying the origin of local thalamic interneurons is challenging because the thalamus does not generate local GABAergic neurons but it does generate projecting GABAergic neurons. The thalamic progenitors that give rise to projecting GABAergic neurons reside in the rostral tier of the proliferative neuroepithelium of prosomere 2, known as pTH-R (Tou et al., 2007). Indeed, the stream of cells derived from this progenitor domain can be distinguished from the neighboring caudal domain of thalamic progenitors (known as the pTH-C), and from prosomere 3, by the expression of post-mitotic markers like *Sox14, Nkx2.2*, and *Tal1* (Jeong et al., 2011). The cells derived from pTH-R become GABAergic projection neurons that populate diencephalic regions, such as the pHB and the IGL. While IGL neurons project to the suprachiasmatic nucleus and other hypothalamic nuclei, pHB axons target the ventromedial prefrontal cortex, the dorsomedial striatum and the nucleus accumbens, and as such, they are involved in mechanisms that regulate mood (Moore et al., 2000; Fernandez et al., 2018; Anastasiades et al., 2021).

An additional challenge is that extra-thalamic sources of local thalamic GABAergic neurons, like the prethalamus and midbrain, also generate other GABAergic neurons. As well as the *Sox14*-negative local interneurons of the thalamus, the proliferative zone of the prethalamus gives rise to many other GABAergic cells. At E10 in mice, the progenitor cells found in the ventricular zone of the prethalamus are characterized by strong expression of transcription factors like *Olig2, Dlx* genes and *Foxd1* (Tou et al., 2007; Blackshaw et al., 2010; Newman et al., 2018; Puelles et al., 2021). Before E14, the prethalamic lineage cells differentiate into neurons, and they start migrating laterally and dorsally to populate the nascent RTN, ZI and vLGN (Ono et al., 2008; Inamura et al., 2011). Almost all of these cells are either local or projecting GABAergic neurons, the latter targeting regions of the thalamus, pretectum and midbrain (Jones, 2007).

### Genetic and activity-dependent factors control the development of thalamic interneurons

The neural tube of rodents exhibits three domains of GABAergic progenitors along its rostro-caudal axis (Achim et al., 2013), each characterized by specific genetic programs with distinct terminal selector genes and giving rise to three broad GABA lineages. The borders of these domains are defined by molecular markers and by secondary organizers. Firstly, the rostral domain expands caudally from the ganglionic eminences, through prosomere 3 up to the ZLI in the diencephalon, where GABAergic differentiation depends on the *Dlx1/2* lineage selector genes (Delogu et al., 2012; Le et al., 2017). Secondly, the ZLI separates the rostral domain from the intermediate domain, which expands caudally up to the isthmic organizer at the midbrain-hindbrain boundary. The intermediate domain includes prosomere 2, prosomere 1 and the midbrain, and GABAergic neurogenesis in the intermediate domain relies on the lineage selector genes *Tal2* and *Gata2* (Virolainen et al., 2012). Finally, the caudal domain spans through the hindbrain and spinal cord, where GABAergic fate is acquired through the expression of lineage selector genes like *Ptf1a* and *Tal1* (Hoshino et al., 2005; Muroyama et al., 2005; Fujiyama et al., 2009). Therefore, the combined mesencephalic and prethalamic origin of thalamic interneurons mean they constitute a population with two broad molecular identities: the *Dlx1/2* (enriched in HO nuclei) and the *Gata2/Tal2* lineage (enriched in FO nuclei) (Figures 3A, B).

The GABAergic cells derived from each lineage acquire their identity during development through different genetic programs. Although the molecular mechanisms that confer GABAergic identity have not specifically been studied in thalamic interneurons, it is likely that each subpopulation of thalamic interneurons unfolds genetic programs according to their site of origin. For instance, GABA precursors in the developing midbrain start expressing *Gata2* and *Tal2* after cell-cycle exit, fate determinants that directly regulate the acquisition of a GABAergic phenotype. These cells activate sustained expression of downstream transcription factors related to the maintenance of a GABAergic identity (*Tal1, Gata3, Six3*, and *Gad1*) and to the correct migration of midbrain precursors (*Sox14)* (Delogu et al., 2012). Indeed, in mice lacking GATA2 or TAL2, GABAergic precursors from the midbrain fail to express genes related to GABA neurotransmission and they switch fate, acquiring a glutamatergic identity (Kala et al., 2009; Achim et al., 2013). However, it remains unclear to what extent midbrain-derived thalamic interneurons share a similar developmental trajectory with other midbrain-derived GABAergic cells. A similar genetic program could also be established for the prethalamic-derived thalamic interneurons and although there are no direct studies on this subpopulation, the developmental program is likely to resemble that of other GABAergic neurons derived from the rostral GABAergic domain, such as cortical and striatal interneurons (Lindtner et al., 2019). As such, the *Dlx1/2* transcription factors may contribute to their GABAergic phenotype, either by directly controlling the expression of the GAD isoforms or by indirectly activating *Dlx5* and *Dlx6* transcription, markers of more mature GABAergic precursors (Cobos et al., 2007; Le et al., 2017).

In addition to intrinsic gene regulatory networks, extrinsic factors also influence the development of thalamic interneurons. In the mouse visual system, different developmental processes are thought to be extrinsically influenced by the input that arrives from the retina, ranging from neurogenesis to network recruitment of interneurons (Golding et al., 2014; Charalambakis et al., 2019; Su et al., 2020). This is evident in animal models where retinal projections are absent or compromised. For example, in anophthalmic mice whose optic nerves were severed at birth and in mice with abnormal spontaneous retinal activity during development, thalamic interneurons accumulate in the upper tiers of the dLGN as opposed to adopting the homogenous distribution throughout the nucleus observed in control mice (Figure 3C; Golding et al., 2014). In these models, the synaptic properties of thalamic interneurons were also affected due to the downregulation of presynaptic and postsynaptic proteins, enhancing the excitability of dLGN neurons and disinhibiting the visual thalamocortical system. Similar results were reported in a transgenic mouse (*Math5*^–/–^) in which the optic tract does not develop, and following binocular enucleation in mice soon after birth (Charalambakis et al., 2019). In both scenarios, the distribution of thalamic interneurons was biased toward the dorsal part of the nucleus, failing to develop both mature intrinsic electrical properties and normal synaptic connectivity with relay neurons.

In addition to the alterations in the distribution of interneurons, there are fewer of these cells in the dLGN of *Math5*^–/–^ mice than in control mice. This reduction in the number of interneurons correlates with abnormally weak FGF15 expression by some astrocytes residing in the visual thalamus (Su et al., 2020). Thus, as in other brain structures, it is likely that the release of FGFs contributes to the recruitment and maturation of inhibitory neurons. Accordingly, genetic ablation of FGF15 impairs the migration of thalamic interneurons into the dLGN and they become misrouted into the somatosensory nucleus. The expression of astrocytic FGF15 in the visual thalamus may be regulated by the SHH released from retinal axons (Deven Somaiya et al., 2022), and the expression of astrocytic FGF15 is reduced in the absence of retinal SHH, decimating the recruitment of interneurons. However, more evidence is needed regarding the interaction between SHH released from retinal axons, the SHH signaling cascades in astrocytes and FGF15 expression. Other effects of SHH might also be at play in these processes and for instance, SHH could exert a broader effect on thalamic astrocytes as it participates in astrocyte specification in brain regions like the retina (Dakubo et al., 2008).

## Concluding remarks

In this review we first focus on the development of excitatory neurons of the thalamus, how they extend their axons and receive inputs from the cortex, and the role of spontaneous activity in the development of these projections. Next, we have delved into the information currently available regarding the development of the other main neuronal cell-type present in the thalamus, local GABAergic interneurons. The evidence compiled in this review establishes the state-of-the-art of the field but also, it poses important questions that need to be addressed. For instance, there are few studies that have investigated how the development of thalamocortical excitatory neurons and local GABAergic interneurons is orchestrated. Moreover, further studies are required to disentangle the precise origin of these local GABAergic neurons, as well as comparative studies using ancient species. It is still unclear what molecular signature determines the thalamic fate of interneurons derived from the midbrain, the prethalamus or other regions, as well as the guidance mechanisms that direct interneurons into the thalamus during development. Finally, since it is now well established that the thalamus presents different patterns of spontaneous activity and that changes in these patterns affect the development of other structures like the sensory cortices, it would be interesting to study the impact of this spontaneous thalamic activity on developing thalamic interneurons.

## Data availability statement

The original contributions presented in this study are included in this article/supplementary material, further inquiries can be directed to the corresponding authors.

## Author contributions

GL-B, FM, and IH-G wrote the manuscript. All authors contributed to the article and approved the submitted version.
